# Characteristics and survival of patients diagnosed with cardiac sarcoidosis: A case series

**DOI:** 10.3389/fmed.2022.1051412

**Published:** 2022-12-13

**Authors:** Tiffany L. Brazile, Melissa Saul, Seyed Mehdi Nouraie, Kevin Gibson

**Affiliations:** ^1^University of Pittsburgh School of Medicine, Pittsburgh, PA, United States; ^2^University of Texas Southwestern Medical Center, Dallas, TX, United States; ^3^University of Pittsburgh and The Dorothy P. and Richard P. Simmons Center for Interstitial Lung Disease, Pittsburgh, PA, United States

**Keywords:** cardiac sarcoidosis, cardiovascular magnetic resonance, inflammation, endomyocardial biopsy, cardiomyopathy

## Abstract

**Background:**

Sarcoidosis is a multiorgan system granulomatous disease of unknown etiology. It is hypothesized that a combination of environmental, occupational, and/or infectious factors provoke an immunological response in genetically susceptible individuals, resulting in a diversity of manifestations throughout the body. In the United States, cardiac sarcoidosis (CS) is diagnosed in 5% of patients with systemic sarcoidosis, however, autopsy results suggest that cardiac involvement may be present in > 50% of patients. CS is debilitating and significantly decreases quality of life and survival. Currently, there are no gold-standard clinical diagnostic or monitoring criteria for CS.

**Methods:**

We identified patients with a diagnosis of sarcoidosis who were seen at the Simmons Center from 2007 to 2020 who had a positive finding of CS documented with cardiovascular magnetic resonance (CMR) and/or endomyocardial biopsy as found in the electronic health record. Medical records were independently reviewed for interpretation and diagnostic features of CS including late gadolinium enhancement (LGE) patterns, increased signal on T2-weighted imaging, and non-caseating granulomas, respectively. Extracardiac organ involvement, cardiac manifestations, comorbid conditions, treatment history, and vital status were also abstracted.

**Results:**

We identified 44 unique patients with evidence of CS out of 246 CMR reports and 9 endomyocardial biopsy pathology reports. The first eligible case was diagnosed in 2007. The majority of patients (73%) had pulmonary manifestations, followed by hepatic manifestations (23%), cutaneous involvement (23%), and urolithiasis (20%). Heart failure was the most common cardiac manifestation affecting 59% of patients. Of these, 39% had a documented left ventricular ejection fraction of < 50% on CMR. Fifty eight percent of patients had a conduction disease and 44% of patients had documented ventricular arrhythmias. Pharmacotherapy was usually initiated for extracardiac manifestations and 93% of patients had been prescribed prednisone. ICD implantation occurred in 43% of patients. Patients were followed up for a median of 5.4 (IQR: 2.4–8.5) years. The 10-year survival was 70%. In addition to age, cutaneous involvement was associated with an increased risk of death (age-adjusted OR 8.47, 95% CI = 1.11–64.73).

**Conclusion:**

CMR is an important tool in the non-invasive diagnosis of CS. The presence of LGE on CMR in a pattern consistent with CS has been shown to be a predictor of mortality and likely contributed to a high proportion of patients undergoing ICD implantation to decrease risk of sudden cardiac death.

**Clinical implications:**

Additional studies are necessary to develop robust criteria for the diagnosis of CS with CMR, assess the benefit of serial imaging for disease monitoring, and evaluate the effect of immunosuppression on disease progression.

## Introduction

Sarcoidosis is a multiorgan system granulomatous disease of unknown etiology. It is hypothesized that a combination of environmental, occupational, and/or infectious factors provoke an immunological response in genetically susceptible individuals, resulting in a diversity of manifestations throughout the body (1). Disease may be more prevalent in women and African Americans and its incidence varies by geographic location (2–4).

In the United States, cardiac sarcoidosis (CS) is diagnosed in 5% of patients with systemic sarcoidosis, however, autopsy results suggest that cardiac involvement may be present in over 50% of patients (5–7). These findings suggest that only a small proportion of patients with CS have clinically significant manifestations that prompted evaluation and led to diagnosis. Most patients diagnosed with CS have a history of sarcoidosis affecting other organ systems, most commonly pulmonary (8–10). Patients with predominant cardiac symptoms may have minimal extracardiac diseases, and therefore, sarcoidosis may not be considered in the differential diagnosis, further contributing to underdiagnosis. CS starts with the development of edema due to inflammation followed by granulomatous infiltration and eventual fibrosis and scarring (11). Cardiac manifestations may include conduction abnormalities, ventricular arrhythmias, heart failure, and sudden cardiac death (1, 8, 10, 12). CS can be debilitating and may significantly decrease quality of life as well as survival. Up to 25% of deaths from sarcoidosis in the United States have been attributed to cardiac involvement, whereas this figure is over 50% in Japan (11). Studies have demonstrated that a lower left ventricular ejection fraction (LVEF) is an important clinical predictor of mortality in patients with CS (13).

Currently, there are no gold standard clinical diagnostic criteria for CS. Multiple guidelines exist and are often discordant, although they involve similar elements, including the presence of clinical and radiological manifestations, non-caseating granulomas on biopsy from another organ system, and no evidence of an alternative etiology (9, 14–16). Endomyocardial biopsy in CS diagnosis is considered high risk and has low sensitivity (20–30%) due the patchy distribution of disease (8). These shortcomings have created a role for non-invasive imaging, such as cardiovascular magnetic resonance (CMR) and fluorodeoxyglucose-positron emission tomography (FDG-PET) to aid in diagnosing CS in conjunction with histopathology results from another organ system (17). The availability of advanced imaging techniques has resulted in increased recognition of underdiagnosed CS (17–19). The prevalence of CS has been reported as 3.7–54.9% with advanced imaging techniques, depending on the technique as well as the population under study (11).

As cardiac sarcoidosis is still considered quite rare, it can be challenging to conduct prospective studies to evaluate diagnostic methods and disease prognosis with various treatments, particularly given the heterogeneity of the disease. Case studies have been helpful in elucidating disease patterns, treatment exposure, and mortality that can impact future studies and management. We conducted a retrospective single center review of patients with biopsy-proven extracardiac sarcoidosis who were diagnosed with CS *via* CMR or endomyocardial biopsy to evaluate their cardiac manifestations, the prevalence of their extracardiac organ involvement, and mortality.

## Methodology

### Study population

Patients with an existing diagnosis of biopsy-proven extracardiac sarcoidosis seen at the Simmons Center for Interstitial Lung Disease at University of Pittsburgh Medical Center between 2007 and 2020 were selected for this review. Patients who underwent imaging with CMR and/or endomyocardial biopsy for the evaluation of CS were screened for eligibility. After exclusion of patients with negative CMR or pathology results, patients who were classified as having CS were selected. All patients were evaluated by a physician at the UPMC Dorothy P. and Richard P. Simmons Center for Interstitial Lung Disease.

### Data collection

Results from CMR and endomyocardial biopsy pathology reports were obtained from the electronic health record. They were independently reviewed for CMR features of CS including late gadolinium enhancement (LGE) patterns and/or increased signal on T2-weighted imaging, and/or the presence of non-caseating granulomas on endomyocardial biopsy. Documentation on lung Scadding stage, extracardiac organ involvement, cardiac manifestations, comorbid conditions, treatment history, and vital status were extracted from the electronic health record.

### Data analysis and definitions

Cases included in our analysis had either a CMR consistent with CS and/or a histopathological diagnosis of CS. CMR findings consistent with cardiac sarcoidosis including the presence of multifocal areas of LGE, subepicardial and midmyocardial LGE suggesting a non-infarct pattern, and extension of LGE across the interventricular septum from the left ventricle to the right ventricle (18, 19). Findings may be supported by the presence of increased T2 weighted signal indicative of regions with increased edema and potentially reflecting reversible inflammation. Pathology reports identifying non-caseating granulomas in myocardial tissue sample without evidence of alternative etiologies were considered positive for CS. Summary statistics were used to evaluate the frequency of different cardiac manifestations, extracardiac organ involvement, comorbid conditions, and management including pharmacotherapy and ICD implantation. Categorical variables are expressed as numbers and percentages (%). Continuous variables are expressed as median (IQR). Patients’ vital status were ascertained using Social Security Death Index. The overall survival of patients was presented from diagnosis date to date of death or last follow up date in their electronic health record. Prognostic odds ratio of disease manifestations and treatment were adjusted for age. We applied univariate logistic regression analysis to calculate the prognostic odds ratio of disease manifestations and treatment. We also used age as a covariate to calculate the age adjusted odds ratio. Hazard ratio of statistically significant predictors in logistic models were also calculated using a multivariable Cox regression analysis. Data analysis was performed with Stata 16.2 (StataCorp, College Station, TX).

## Results

We identified 44 unique patients with evidence of CS out of 246 CMR reports and 9 endomyocardial biopsy pathology reports. The median age of the cohort was 55 years, 60% were male and 25% were Black. The majority of patients (73%) had pulmonary manifestations classified according to Scadding stage (Table 1). The next most common extracardiac manifestations were hepatitis or elevated transaminases (27%), cutaneous involvement (23%), urolithiasis (20%), hypercalcemia (16%), joint involvement (18%), and peripheral neuropathy (16%). Heart failure was the most common cardiac manifestation affecting 59% of patients. Of these, 39% had a documented left ventricular ejection fraction (LVEF) of < 50% on their diagnostic CMR. The median (IQR) of LVEF was 56% (40–63).

**TABLE 1:** Demographic and clinical characteristics of patients with cardiac sarcoidosis (*N* = 44).

Characteristic	Results
Age, median (IQR)	55 (46–61)
Male, *n* (%)	26 (59)
Black, *n* (%)	11 (25)
**Initial lung Scadding stage, *n* (%)**	
0	12 (27)
1	7 (16)
2/3	19 (43)
4	6 (14)

**FIGURE 1:**
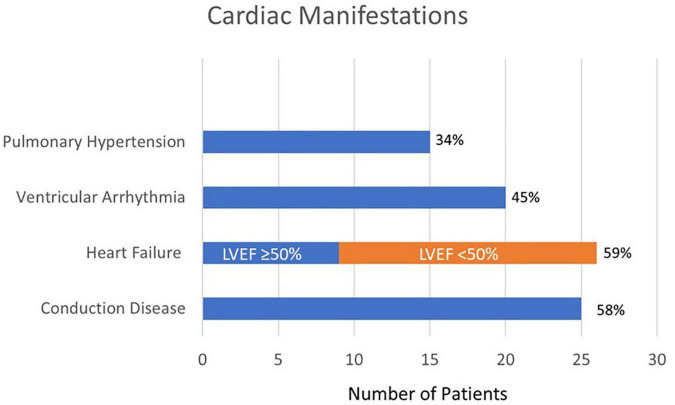
Cardiac manifestations of patients with cardiac sarcoidosis (*N* = 44).

The observed cardiovascular manifestations in our patients are displayed in Figure 1. Fifty eight percent of patients had a conduction disease, including 5% with some degree of AV nodal block, 39% with dysfunction in the His-Purkinje system as manifested as bundle branch blocks, and 14% had a combination of both AV nodal block and bundle branch block. Heart failure was diagnosed in 59% of patients, 65% of whom had an ejection fraction of < 50%. Forty-four percent of patients had documented ventricular arrhythmias. One third of patients had a documented history of pulmonary hypertension in addition to their other cardiac findings. More than a quarter of patients (27%) had a history of coronary artery disease, which was based on history of myocardial infarction or the presence of coronary calcifications on imaging.

Therapeutic interventions received by patients during the study period are detailed in Table 2. Nearly all patients (93%) were on prednisone at some point during their treatment course. The most commonly used steroid-sparing agent was methotrexate (75%). Forty three percent of patients underwent ICD implantation.

**TABLE 2:** Pharmacological treatment of patients with cardiac sarcoidosis (*N* = 44).

Medication	*n* (%)
Prednisone	42 (93)
Methotrexate	33 (75)
Infliximab	14 (32)
Azathioprine	5 (11)
Mycophenolate mofetil	5 (11)
Adalimumab	3 (7)

**FIGURE 2:**
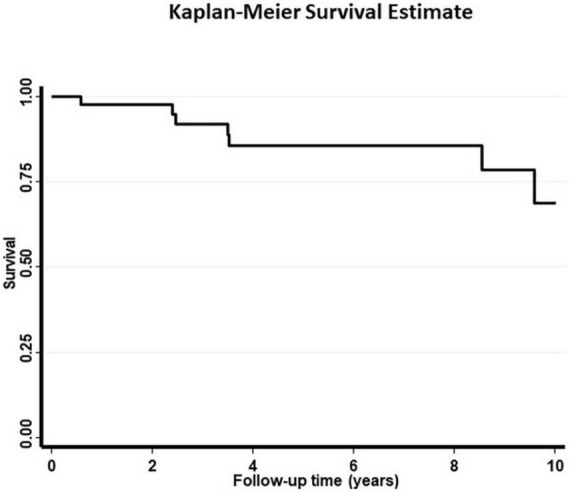
The 10-year survival curve for 44 patients with cardiac sarcoidosis.

Patients were followed up for a median of 5.4 (IQR: 2.4–8.5) years. The 10-year survival was 70% (Figure 2). In addition to age, cutaneous involvement was associated with an increased risk of death (age adjusted OR = 8.47, 95% CI = 1.11–64.73, Table 3). LVEF < 50% (age adjusted OR = 1.1, *p* = 0.9) was not associated with significantly higher risk of mortality. In unadjusted analysis the presence of conductive disease was a risk factor of mortality which was not statistically significant after adjusting for age. Prednisone treatment was also a protective factor in unadjusted analysis (Table 3). In a survival analysis using a Cox regression model, both age (HR = 1.15 per year, 95% CI = 1.04–1.27, *p* = 0.007) and cutaneous involvement (age adjusted HR = 6.90, 95% CI = 1.33–35.95, *p* = 0.02) were associated with poor survival.

**TABLE 3:** Association between important baseline factor and mortality (*N* = 44).

	Unadjusted		Age adjusted	
	OR (95% CI)	*P*-value	OR (95% CI)	*P*-value
Age	**1.11 (1.01–1.22)**	**0.033**	NA	
Male	2.09 (0.42–10.33)	0.4	2.82 (0.50–15.84)	0.24
Initial lung Scadding stage >1	2.33 (0.47–11.50)	0.3	2.16 (0.39–11.84)	0.37
Skin involvement	4.69 (0.99–22.26)	0.052	**8.47 (1.11–64.73)**	**0.040**
Heart failure	4.49 (0.69–29.06)	0.12	4.28 (0.62–29.41)	0.14
Conduction abnormalities	**18.94 (1.02–352.73)**	**0.049**	15.92 (0.83–306.01)	0.07
Pulmonary hypertension	3.97 (0.89–18.11)	0.08	3.62 (0.71–18.32)	0.12
Prednisone	**0.11 (0.01–0.98)**	**0.048**	0.14 (0.02–1.31)	0.09

Bolded results have a *p*-value < 0.05.

## Discussion

Our single center case series describes a heterogenous population of patients with biopsy-proven extracardiac sarcoidosis who were diagnosed with CS by CMR and/or endomyocardial biopsy with a median follow up of 5 years. Our overall 10-year survival rate was 70%. This is lower than reported range of 70–93% for 10-year survival rates (20–22). It is challenging to directly compare these rates due to differences in methodology such as inclusion criteria, statistical analysis, and outcomes of interest. The retrospective study by Kandolin et al. selected patients based on the development of cardiac symptoms who were found to have sarcoidosis rather than patients with an established diagnosis of sarcoidosis (20). This may have resulted in a significant proportion of their patients (65%) having clinically isolated CS, which may have detected CS at an earlier stage. Additionally, the outcome of interest was 10-year cardiac survival, whereas our study focused on overall survival. In a study by Zhou et al. the 10-year survival rate was 93.4% (21). Only a small proportion of patients (31.5%) had LGE on CMR in contrast to the majority of patients in our study, which may suggest detection of disease at an earlier stage or lead time bias. Furthermore, nearly 60% of patients had an ICD implanted compared with only 43% in our study. This difference in mortality suggests a protective effect of ICD implantation. Furthermore, while ICD implantation was not an independent risk factor for mortality in our patients, the unadjusted OR of conduction abnormalities suggest that it is a prognostic factor in patients with CS. Furthermore, the median age at diagnosis in this cohort was 46, nearly a decade younger than the patients in our study. A similar survival rate was reported by Cacoub et al. in patients with a median age of 40 (22). The impact of other comorbid conditions, which are not consistently reported in other studies, such as hypertension, obesity, diabetes, and smoking may further help to explain differences in 10-year survival among these populations. The significant difference in age as well as stage of disease at diagnosis may account for some of the differences observed in 10-year survival.

Conduction disease is highly prevalent in patients with CS, which may be due to a high frequency of disease manifestation in the basal septum (11, 17). The prevalence of AV nodal conduction disease was slightly lower in our population than in other published studies (19% vs. 25–30%). The prevalence for His-Purkinje system disease in our study was slightly higher at 39% compared to the 12–32% reported in other studies. It is possible that the increased prevalence of His-Purkinje disease in our population is attributable to a high proportion of patients (93%) having coexisting CAD and may not be directly due to CS.

Ventricular arrhythmias were more common in our case series at 44% than reported in previous studies at 23–40%. Prior studies have noted that ventricular arrhythmias may be the first presentation in up to 40% of patients with CS (23, 24). As the ability to diagnose CS improves, it is likely that the incidence, and therefore, prevalence of ventricular arrhythmias attributed to CS will increase. Ventricular arrhythmias may also be secondary to ischemia from CAD and not CS, which can be challenging to discern and will vary among populations. Studies show that patients suffering from ventricular arrhythmias secondary to CS may be less responsive to antiarrhythmic pharmacotherapies (25). As a result, these patients often require ICD implantation.

ICD implantation appears to be protective from sudden cardiac death in patients with CS regardless of whether they meet the typical indications for placement, such as ventricular tachycardia/fibrillation and/or low LVEF (26, 27). As ICD placement is a shared decision between the patient, cardiologist and pulmonologist, not all patients who meet criteria for ICD implantation will receive one (28, 29). Only 43% of patients in our study received an ICD, which is lower than in the study by Zhou et al. (21).

The prevalence of heart failure in this study is consistent with reported prevalence of 25–75% among other case series (30, 31). Left ventricular remodeling occurs in the setting of granulomatous inflammation and scarring. The degree of left ventricular systolic dysfunction, as measured by LVEF, is reported to be the most powerful predictor of mortality (13, 32), however, our study did not demonstrate this. Instead, our study showed that patients with conduction disease were nearly 19 times less likely to survive than those without such manifestations. More studies are needed to elucidate the use of advanced imaging as a prognostic factor in CS and its relationship to clinical cardiovascular manifestations.

While 33% of the cases in our study have documented pulmonary hypertension, reported prevalence has been highly variable. Depending on the population studied, pulmonary hypertension ranges from 6% in outpatient study to over 70% in patients listed for lung transplantation (33, 34). Furthermore, 40–60% of patients with sarcoidosis related pulmonary hypertension do not have evidence of overt lung parenchymal involvement such that Scadding stage may be discordant with disease severity and risk of death (35, 36). In this population, pulmonary hypertension may instead be caused by sarcoidosis related left ventricular systolic dysfunction, extrinsic compression of the pulmonary vasculature by mediastinal lymphadenopathy, chronic hypoxic vasoconstriction leading to cor pulmonale, and/or remodeling of the pulmonary vasculature (33, 34). In our cohort, 20 underwent RHC of whom 7 were diagnosed with pulmonary hypertension. However, there were 8 additional patients who were suspected to have pulmonary hypertension based on transthoracic echocardiography or stigmata on computed tomography of the chest. Only 3 of the 7 patients diagnosed with pulmonary hypertension *via* right heart catheterization were prescribed pulmonary artery vasodilators due to the presence of precapillary pulmonary hypertension. The remainder were managed through treatment of their underlying disease process, most commonly left-sided heart disease in the setting of sarcoidosis.

The frequency of extracardiac manifestations in our case series was in line with other studies. We were unable to capture isolated CS based on our inclusion criteria. Over 70% of patients in our cohort with CS have concomitant pulmonary sarcoidosis, which is consistent with other studies (9, 10). Cutaneous manifestations of sarcoidosis, which occur in 25–33% of patients, are heterogeneous with prognosis varying by subtype (37, 38). Specifically, lupus pernio and plaques have been associated with increased mortality, whereas erythema nodosum, Lofgren syndrome, maculopapular, and nodular lesions are associated with low mortality risk. Of the 10 patients with cutaneous manifestations in our study, only one had plaques and none had evidence of lupus pernio. As a result, it is challenging to explain the increased mortality risk in this subset of patients based on the presence of cutaneous manifestations alone.

Early administration of corticosteroids is the mainstay of treatment with subsequent tapering to tolerance, despite the lack of randomized control trials (22, 39). The majority of patients in our study received steroids (93%). Many transitioned to a steroid-sparing agent later in their disease course since maintaining adequate levels of corticosteroids to suppress inflammation and scarring is difficult over the long term due to its side effects (22). As studies exploring the use of steroids are retrospective, their effect on the natural history of CS is unknown. It is challenging to draw conclusions about steroid dosing or duration due to the heterogeneous nature of observational studies. A study by Kandolin et al. that evaluated transplant-free cardiac survival suggested that long-term outcomes are independent of initial steroid dosing and timing (20). The long-term benefits of steroid use, however, may depend on the presence and severity of left ventricular systolic dysfunction, one of the strongest prognostic indicators for survival (40). Retrospective studies have demonstrated that even with corticosteroid therapy, there is limited evidence of improvement when the LVEF is less than 30% (31, 32). Thus, corticosteroid treatment may be of benefit in early-to-mid stage disease prior to the development of fibrosis. Steroid sparing agents are increasingly used for long term therapy, but their safety and efficacy has yet to be established. The Cardiac Sarcoidosis multi-center randomized controlled trial (CHASM CS- RCT) is currently underway to compare different regimens (41).

It is unclear if we can distinguish survival benefit from the use of steroids in patients who are also receiving guideline directed medical therapy for heart failure. The management of ventricular tachyarrhythmias secondary to CS is difficult as inducibility may vary between active and quiescent phases of the disease and there are no studies evaluating the use of antiarrhythmics in CS (39). Other studies demonstrate improvement in AV nodal conduction with steroid therapy (13, 20). One study of symptomatic cardiac sarcoidosis patients concluded that the combination of steroids plus other immunosuppressive therapies may reduce the risk of relapse compared with steroids alone (42). In our study, the presence of an ICD did not significantly impact mortality risk.

The generalizability of the results from our case series is limited as it is from a single Sarcoidosis Center. The majority of the patients were diagnosed with CS *via* CMR; however, no FDG-PET images were available for comparison as this imaging modality is not currently available at our institution. Based on the inclusion criteria of our case series, namely biopsy-proven extracardiac sarcoidosis, we were unable to capture cases of isolated CS, for which the prevalence remains unknown. Currently, no protocol exists to screen for CS in the absence of cardiovascular symptoms in patients with sarcoidosis, although it may remain clinically silent for some time. Similarly, the value of repeat imaging in patients with cardiovascular symptoms with a negative initial scan has yet to be evaluated.

This case series focused on cardiac manifestations and extracardiac organ involvement in patients with CS to better understand the presentation of disease, the therapies most commonly prescribed, and frequency of advanced therapy with ICD implantation. Additional studies are necessary to develop robust criteria for the diagnosis of CS with advanced imaging techniques, to directly compare CMR and FDG-PET, assess the benefit of serial imaging for disease monitoring, and evaluate the effect of immunosuppressive therapies on disease progression. To date, it has been challenging to develop these types of studies given the low prevalence of disease, the multitude of confounding factors associated with treatment, and the reluctance to modify or discontinue treatment once remission is achieved.

## Data availability statement

The datasets presented in this article are not readily available because due to IRB restrictions, data is not available for outside investigators. Requests to access the datasets should be directed to SN.

## Ethics statement

The University of Pittsburgh IRB reviewed and approved “STUDY20010219”. Written informed consent for participation was not required for this study in accordance with the national legislation and the institutional requirements.

## Author contributions

TB, MS, SN, and KG contributed to the conception and design of the study. MS organized the database. TB abstracted electronic medical records and wrote the first draft of the manuscript. SN performed the statistical analysis. SN and KG wrote sections of the manuscript. All authors contributed to the manuscript revision, read, and approved the submitted version.
